# The Slavcleft: A Three-Center Study of the Outcome of Treatment of Cleft Lip and Palate Considering Palatal Shape

**DOI:** 10.3390/jcm12185985

**Published:** 2023-09-15

**Authors:** Tereza Petrova, Andrzej Brudnicki, Magdalena Kotova, Wanda Urbanova, Ivana Dubovska, Petra Polackova, Iva Voborna, Piotr S. Fudalej

**Affiliations:** 1 Department of Stomatology, 3rd Faculty of Medicine, Charles University and Kralovske Vinohrady University Hospital, 10034 Prague, Czech Republic; 2 Institute of Dentistry and Oral Sciences, Faculty of Medicine and Dentistry, Palacký University Olomouc, 77900 Olomouc, Czech Republic; 3 Department of Pediatric Surgery, Institute of Mother and Child, 01-211 Warsaw, Poland; 4 Faculty of Medicine in Pilsen, Charles University, 323 00 Pilsen, Czech Republic; 5 Department of Orthodontics and Dentofacial Orthopedics, University of Bern, 3012 Bern, Switzerland; 6 Department of Orthodontics, Jagiellonian University Cracow, 31-155 Krakow, Poland

**Keywords:** cleft lip and palate, palatal shape, geometric morphometrics, surgical protocol

## Abstract

The degree of deviation of palatal shape from the norm may reflect facial growth disturbance in cleft lip and palate (CLP). The objective of this study was to compare the palatal morphology in children treated with different surgical protocols. Palatal shape was assessed with geometric morphometrics (GM) including Procrustes superimposition, principal component analysis (PCA), and permutation tests with 10,000 permutations, in 24 children treated with two-stage repair with a late palatoplasty (Prague group; mean age at assessment 8.9 years), 16 children after two-stage repair with early palatoplasty (Bratislava group; mean age 8.2 years), and 53 children treated with a one-stage repair (Warsaw group, mean age 10.3 years). The non-cleft control group comprised 60 children at 8.6 years. The first five principal components (PCs) accounted for a minimum of 5% of the total shape variability (65.9% in total). The Procrustes distance was largest for the Prague vs. Control pair and smallest for the Prague vs. Bratislava pair. Nonetheless, all intergroup differences were statistically significant (*p* < 0.01). One can conclude that variations in palatal shape roughly correspond to cephalometric and dental arch relationship findings from prior research. Among the children who underwent a one-stage repair of the complete cleft, their palatal morphology most closely resembled that of the non-cleft controls. Conversely, children who received late palatoplasty exhibited the greatest degree of deviation.

## 1. Introduction

The existing data from both clinical and experimental research strongly suggest that palatal surgery plays a significant role in causing maxillary growth disturbances among patients with cleft lip and palate (CLP). As early as the late 1960s, Kermenak et al. conducted experiments by removing mucoperiosteal flaps on the palatal surface in young beagle dogs to simulate the clinical situation after cleft palate repair [1]. This approach resulted in growth disturbances similar to those observed after surgical cleft closure in children. Subsequent experimental studies [2,3] have emphasized the importance of wound contraction and scarring in surgical wounds. Specifically, scar tissue attaching to the palatal bone and the periodontal fiber system inhibits sutural growth and interferes with the normal development of the dentition [4].

Over the years, various researchers have made efforts to modify cleft palate surgery to prevent potential growth disturbances. Single-stage cleft palate repair techniques, such as the von Langenbeck [5,6], Veau–Wardill–Kilner push-back [5], and Bardach two-flap techniques [7,8] have been recommended. For two-stage repair, the Schweckendiek [9] and Delaire [10] techniques have been suggested. Additionally, intervelar veloplasty in the soft palate by re-orientation of the levator muscle has been advocated by Kriens [11] and Sommerlad [12], while the Furlow Z-plasty technique [13] has been used to improve soft palate length. Despite these efforts, completely eliminating palatal scarring has proven to be a challenge. The repair of the cleft palate in children with CLP is still associated with risk of long-term unfavorable maxillofacial growth.

The consequences of palatoplasty were considered during the development of the Eurocran Index (EI). This index is a modification of the GOSLON Yardstick and 5YO index [14] and serves to evaluate dental arch relationships using a 4-point scale, along with assessing palatal morphology using a 3-point scale. Notably, the Eurocran Index seems to be the only index that simultaneously assesses two critical components: the occlusal relationship in all three planes of space, including the displacement of the lesser segment on the cleft side, and the palatal morphology [15]. The EI was recently utilized in the Scandcleft trial, a large multicenter randomized clinical trial focusing on primary surgeries for cleft lip and palate [16]. This employment of the EI allowed for the comprehensive comparison of dental arch relationships at 5, 8, and 10 years, providing valuable insights into the long-term effects of cleft surgery. However, it should be noted that the reliability of evaluating palatal morphology with the EI can pose challenges because of the variable palatal anatomy and scarred tissues. Despite this limitation, the use of the EI in such a substantial project underscores the evident importance of assessing the palatal shape.

The Slavcleft project is a collaborative research initiative focused on investigating the treatment outcomes of patients with cleft lip and palate in three Central European cleft centers—Warsaw, Prague, and Bratislava, utilizing distinct treatment protocols. Previous findings [17,18] have indicated that maxillofacial growth was relatively more favorable in children treated in Warsaw, compared to those treated in Bratislava and Prague. For instance, the maxillary prominence, described by the Sella–Nasion-point A (SNA) angle, was found to be nearly 3° larger in Polish patients compared to their Slovakian peers; the maxillomandibular relationship, assessed by the point A-Nasion-point B (ANB) angle, was nearly 3° larger in the Polish group than in the Czech group [17]. Moreover, the evaluation of dental arch relationships using the Goslon yardstick showed better outcomes in Polish patients than in Czech patients [18]. However, it is important to note that palatal morphology was not assessed in these patients. Hence, it can only be assumed that the degree of deviation of palatal shape from the norm may reflect the facial growth disturbance observed in the previous investigation. Therefore, the objective of this study was to compare the palatal morphology in children who were treated in Warsaw, Prague, and Bratislava, as well as non-cleft controls. The research hypothesis (HR) was that all groups would exhibit differences in palatal morphology.

## 2. Materials and Methods

### 2.1. Subjects

The study used digital models of the maxilla of children without the cleft (Control group) and with complete unilateral cleft lip and palate (UCLP) who were treated in cleft centers located in Warsaw (Poland), Prague (Czech Republic), and Bratislava (Slovakia). These centers employed different surgical protocols, as detailed in the first part of the Slavcleft study [17].

In summary, the Warsaw Cleft Center, affiliated with the Institute of Mother and Child (IMC), treated 53 patients with a one-stage cleft repair at 8 months. The mean age of records taken was 10.6 years (SD = 1.4) with a gender proportion of 69.8% males and 30.2% females.

The Prague Cleft Center, affiliated with the Kralovske Vinohrady University Hospital, treated 24 children with a two-stage repair, performing lip repair at 7 months and palatoplasty at 36 months. The mean age of records taken was 8.9 years (SD = 0.8), with a gender proportion of 70.8% boys and 29.2% girls.

The Bratislava Cleft Center, affiliated with the Clinic of Plastic and Reconstructive Surgery, Comenius University, treated 16 children with a two-stage repair, performing lip repair at 4 months and palatoplasty at 11 months. The mean age of records taken was 8.2 years (SD = 1.3), with a gender proportion of 68.8% boys and 31.2% girls.

The control group consisted of 60 children (25 boys and 35 girls) with a mean age of 8.6 years (SD = 1.2) at the time of making plaster models. They met the inclusion/exclusion criteria, which included being healthy, having Class 1 malocclusion, no cross-bite, no previous orthodontic treatment, no multiple and/or advanced caries, no tooth agenesis/supernumerary teeth, and no cleft lip and/or palate and other congenital facial syndromes.

### 2.2. Methods

**Figure 1:**
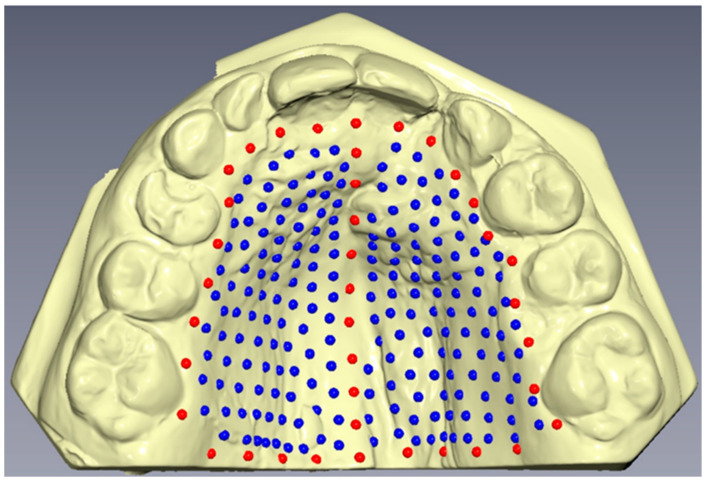
Graphical representation of fixed landmarks (red) and semi-landmarks (blue) drawn on the palatal surface of digital casts.

Good quality plaster casts of the maxilla were scanned using the Trios intraoral scanner (3Shape A/S, Copenhagen, Denmark) and iTero^TM^ intraoral scanner (Align Technology Inc., San Jose, CA, USA), and the resulting meshes were saved as STL files. A total of 239 landmarks were then digitized on each digital model using the Viewbox version 4 program (dHAL software, Kifissia, Greece) (Figure 1). The initial placement of 39 landmarks, referred to as fixed landmarks, involved 9 landmarks along the midsagittal suture, 21 landmarks forming a perimeter confine along the dental arch and passing apical to the gingival sulci of each tooth, and 9 landmarks establishing a posterior confine—a curve passing from the distal of the first permanent molars, perpendicular to the midsagittal line. The remaining landmarks, referred to as semi-landmarks, were uniformly placed on the palatal surface within the boundaries defined by the fixed landmarks. These semi-landmarks were then iteratively slid to minimize bending energy, projected back onto the palatal surface, and further slid three times. As a result, all landmark positions were considered homologous across the subjects.

All digitizations of maxillary models were performed by the same operator to ensure consistency and reduce potential operator-related biases.

### 2.3. Statistical Analysis

The homologous landmark configurations underwent generalized Procrustes superimposition to standardize the palatal shape data. The resulting Procrustes landmark coordinates were then subjected to principal component analysis (PCA), a dimensionality reduction method that retains most of the original shape information while transforming it into a smaller set of principal components (PCs). To determine the number of principal components containing meaningful shape information, the broken-stick criterion was applied. Differences between the study groups and between males and females were evaluated using permutation tests with 10,000 permutations, with statistically significant differences considered for *p*-values < 0.05.

### 2.4. Method Error

To assess the reliability of the digitization process, 20 randomly selected maxillary models were re-digitized at least 1 month apart. The method error was quantified as the Procrustes distance between the repeated digitizations, relative to the total shape variance.

## 3. Results

### 3.1. Demographic Data and Method Error

The demographic characteristics of the sample are summarized in Table 1. The Warsaw group consisted of the oldest children at the time of evaluation, while the other groups comprised children at comparable ages, with a difference of approximately 1.5 years. Palatal shape exhibited similarity between girls and boys, irrespective of the presence of a cleft or group. Therefore, both sexes were analyzed collectively.

The method error accounted for 10.8 percent of the total shape variance.

### 3.2. Procrustes Superimposition and PCA

**Figure 2:**
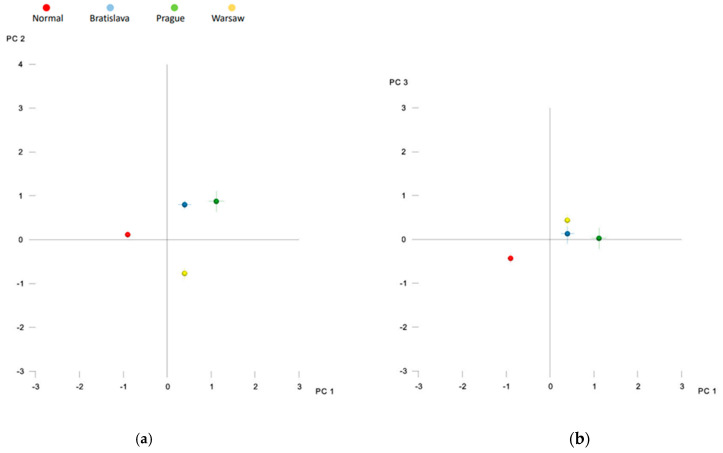
Distribution of group averages in the shape space described by (**a**) principal component 1 and 2 (PC1 and PC2) and (**b**) principal component 1 and 3 (PC1 and PC3).

**Figure 3:**
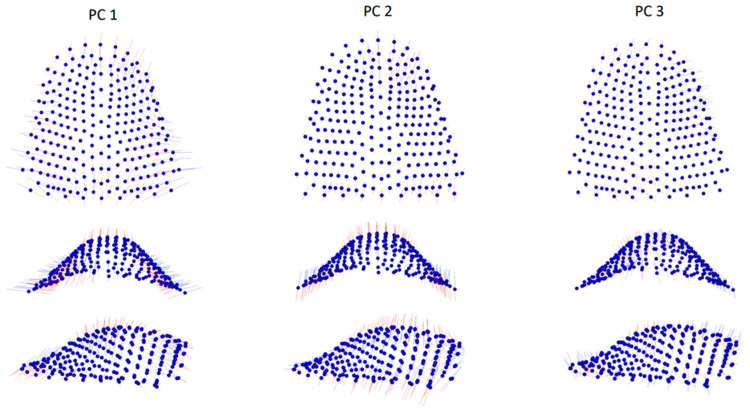
Graphic depiction of the first three principal components (PCs) of the palate from the three views. Red lines: −3 standard deviation, blue lines: +3 standard deviation.

The broken-stick criterion indicated that the first twenty principal components (PCs) captured significant shape variation, explaining 91.9% of the total shape variability (see Supplementary Table S1). Among these, the first five PCs accounted for a minimum of 5% of the total shape variability, with PC1 representing 23.9%, PC2 18.5%, PC3 10.9%, PC4 7.4%, and PC5 5.3% (65.9% in total). The distribution of group means in shape space is depicted in Figure 2a,b. PC1 primarily represented variations in width and length and also described variations in palatal vault height (excluding the region adjacent to teeth). Thus, subjects with narrower palates also had longer palates and higher palate vaults. PC2 mainly depicted variations in the height of the entire palate region, while PC3 showed cleft side-related variations across all three dimensions, as illustrated in Figure 3.

### 3.3. Inter-Group Differences

**Figure 4:**
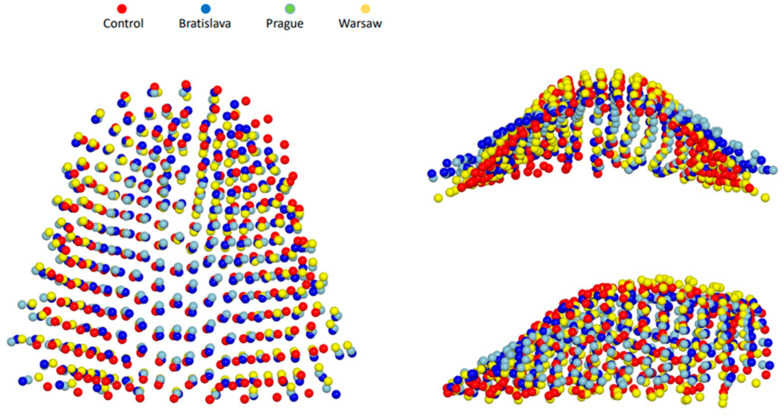
Graphic depiction of superimposed consensus shapes of control (red spheres), Bratislava (dark blue spheres), Prague (light blue spheres), and Warsaw (yellow spheres) groups from the three views.

**Figure 5:**
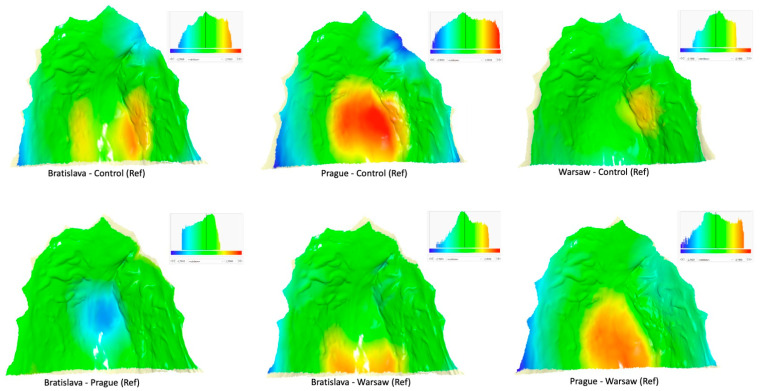
Heat maps with pairs of consensus shapes of the groups. Ref denotes which group was used as reference. Colors describe which parts of palate of the consensus shape of the respective group is in front of the Reference (red, yellow), close to the Reference (green), or behind the Reference (blue). A yellowish color at some parts of the margin denotes overhang regions for which heat maps were not calculated.

Table 2 presents the differences in shape space between the groups. The Procrustes distance was largest for the Prague vs. Control pair and smallest for the Prague vs. Bratislava pair. Nonetheless, all intergroup differences were statistically significant. Figure 2a,b indicate that subjects without a cleft differed from all cleft groups primarily along the PC1 axis, while the Warsaw group differed from the Prague and Bratislava groups along the PC2 axis. Prague and Bratislava groups also exhibited some differences relative to the PC1 axis, but the distinction was smaller compared to the differences between the Control and other cleft groups. The superimposition of consensus shapes of the groups (Figure 4) and heat maps (Figure 5) revealed that all cleft groups had narrower palatal vaults but wider palates in the region adjacent to teeth. Subjects from Warsaw had the highest palatal vault, while subjects from Bratislava and Prague had shallower palatal vaults compared to both Warsaw and Control groups.

## 4. Discussion

Slavcleft is a collaborative research project focused on investigating the treatment outcomes of patients with cleft lip and palate in cleft centers in Central European countries, utilizing distinctive treatment protocols. These protocols include two-stage closure of the cleft lip and palate with palatoplasty at 11 months in Bratislava, two-stage closure with delayed palatal repair at 36 months in Prague and one-stage closure of the entire cleft at 9 months in Warsaw. Previous findings from the Slavcleft project suggest that certain growth outcomes were more favorable in children treated in Warsaw compared to those treated in Bratislava and Prague. In this study, we focused on analyzing palatal shape, as the repair of the cleft palate is assumed to have the largest effect on future growth of this area. We assumed that significant deviation in palatal shape from normal growth is associated with poorer growth outcomes in children with cleft lip and palate.

As mentioned above, the one-stage repair of the entire cleft used in Warsaw produced relatively good results compared to other Slavcleft participants. Polish children treated in Warsaw showed more favorable antero-posterior position of the maxilla and dental arch relationship when compared to their Czech and Slovakian peers. Additionally, the need for orthognathic surgery, defined as Goslon 4 and 5 grades, was significantly less after the one-stage protocol in Warsaw than after the protocols used in Bratislava and Prague. Only 16% of children treated in Warsaw were graded as Goslon 4 or 5, whereas the proportion of Goslon 4 or 5 grades was 37% and 45% in the Bratislava and Prague samples, respectively. The results of the palatal shape analysis performed in the current study align with these findings, showing that in comparison with children without a cleft, the palatal shape in the Warsaw group was the least deviant from the norm, while the degree of deviation from the normal shape was largest in the Prague group. Heat maps imply a slight constriction of the palatal vault on the cleft side and some restriction of the vertical growth in the region of the cleft in Warsaw group, whereas palatal vault was significantly shallower with considerable restriction of the vertical growth in the region of the cleft in Prague group.

The one-stage closure of UCLP performed in Warsaw follows a specific sequence, beginning with the soft palate, followed by the hard palate, and concluding with the restoration of the upper lip continuity. This approach allows easier access to the operated region when the cleft lip is still unrepaired. This refined surgical technique of palatoplasty has been developed over the last 30 years to avoid leaving any open wounds on the surface of the hard palate bone. Thanks to it, scar formation on the surface of the palate is minimized. Although it is challenging to isolate the influence of specific components of the treatment protocol for cleft lip and palate on the final outcome, it can be hypothesized that the atraumatic one-stage repair of the cleft performed by highly experienced surgeons, as is the case in Warsaw, significantly contributes to relatively small deviation of palatal shape.

It was surprising to observe significant impairment of palatal morphology in Czech patients, with a shallow palatal vault and notable impediment in vertical growth within the cleft area. This outcome occurred in spite of the fact that the palatal repair was delayed until the age of 3 years to avoid potential growth-impairing effects of early palatoplasty. Cleft teams employing a delayed approach have argued that patients who undergo delayed hard palate repair may exhibit better maxillofacial growth compared to those who undergo early palatoplasty. This notion was supported by a large meta-analysis of treatment outcomes evaluated with the Goslon Yardstick in 1234 patients, which indicated that delayed palatoplasty was associated with better dental arch relationships [19]. Subsequent systematic reviews, however, produced conflicting results and did not provide definitive evidence to either support or refute the idea that postponing hard palate surgery brings benefits for maxillary growth [20,21]. It is important to note that the recent results of the Scandcleft trial [22] were not included in these reviews. These results showed that timing (including postponement of hard palate closure until 3 years) did not significantly impact growth outcomes in UCLP. As a result, it appears that other factors, such as the participation of low-volume surgeons or a surgical technique, may have had an unfavorable effect on palatal shape in patients from the Prague cleft center.

The average age of palatal shape assessment differed among the groups in our study. Although the assessment was conducted prior to the growth spurt, we acknowledge the potential influence of developmental changes during this period on the outcomes. However, the research by Primožič et al. [23] and Yang et al. [24] suggest that alterations in palatal dimensions between the ages of 8 and 10 are limited. Given these findings, it is reasonable to assume that the disparities in assessment age among our study groups likely exerted an insignificant impact on our results.

We utilized GM to study palatal shape in our sample. GM, which has been continuously refined over the past 30 years, involves the use of spatial coordinates of landmarks and semi–landmarks—specific anatomical points identified on biological structures. Various statistical and geometric tools are applied to analyze and interpret shape variations, with visualization and identification of geometric relationships among individuals or groups being key strengths [25]. However, GM has certain limitations that should be considered. One limitation is that it assumes all shape variables are geometrically independent, meaning changes in one variable do not affect others. In reality, landmarks are biologically linked, and closely adjacent landmarks cannot vary independently. Increasing landmark density in a region leads to higher weighting of that region in multivariate distances and statistics, affecting the quantification of overall shape differences between groups. Plausible biological or mechanical models are needed for meaningful interpretation of multivariate distances. Another challenge arises from the sheer number of variables in GM. Measurement errors for each coordinate, along with actual anatomical form differences, contribute to the Procrustes distance between two configurations. Increasing the number of measured landmarks introduces more measurement errors and increases the Procrustes distance, making the magnitude of shape differences dependent on the number of measured variables. Moreover, it is worth noting that presenting a clear biological interpretation of variability identified through individual principal components can be challenging. This limitation stems from the fact that principal component analysis is a purely mathematical technique and lacks inherent consideration of biological or clinical principles. As a consequence, when examining variability in the sample along the PC1 axis, it becomes evident that the observed variations are not confined to any specific anatomical region. Instead, the variability is dispersed across different regions, each exhibiting distinct degrees of variability along the PC1 axis. As a result, caution should be exercised when interpreting geometric morphometric results in our study and other similar investigations, considering these inherent limitations.

### 4.1. Limitations

This study has some limitations that should be considered when interpreting the results. Firstly, one significant limitation is the non-consecutive inclusion of patients in our cleft groups, which could introduce selection bias and may not fully represent the entire cleft population. Future studies should aim to include consecutive patients to enhance the generalizability of the findings. Another limitation is the method error, accounting for 10.8% of the total shape variability. This level of method error is approximately 40% higher than in previous studies that assessed palatal shape. It is important to consider that previous studies mainly focused on patients without clefts, who typically have more regular palatal morphology. The increased method error in our study might be due to the inherent challenges and variations in analyzing palatal shape in individuals with clefts, where the presence of cleft-related deformities can complicate the analysis process. Two scanners—Trios and iTero—were used to digitize plaster models which were further analyzed. This might have introduced some error in the investigation which was not evaluated. Finally, it is important to note that the assessment was conducted before the completion of growth and treatment. Therefore, additional evaluation after the age of 16 (i.e., when the growth of the facial skeleton is complete) is necessary to gain a more comprehensive understanding of the long-term effects and outcomes of the treatments.

### 4.2. Conclusions

Given the limitations of this study, one can conclude that variations in palatal shape roughly correspond to cephalometric and dental arch relationship findings from prior research. Among the children who underwent a one-stage repair of the complete cleft, palatal morphology most closely resembled that of non-cleft controls. Conversely, children who received late palatoplasty exhibited the greatest degree of deviation. Nonetheless, identifying the factors accountable for these outcomes was unattainable within the scope of this retrospective study.

## Figures and Tables

**Table 1:** Demographic description of the groups.

	Age in Years at Palatoplasty (Mean, SD, min, max)	Age in Years at Collection of Models (Mean, SD, min, max)
**Prague**		
males (n =17)	2.6; 0.5; 1.5–3.3	9; 0.8; 7.3–10.1
females (n = 7)	3.9; 1.2; 2.7–6.4	8.7; 0.9; 7.3–10
males and females (n = 24)	3; 1; 1.5–6.4	8.9; 0.8; 7.3–10.1
**Bratislava**		
males (n = 11)	0.9; 0.4; 0.6–1.8	8.3; 1.3; 6.1–10.3
females (n = 5)	0.9; 0.4; 0.7–1.7	8; 1.3; 6.3–9.5
males and females (n = 16)	0.9; 0.4; 0.6–1.8	8.2; 1.3; 6.1–10.3
**Warsaw**		
males (n = 37)	0.6; 0.1; 0.4–1	10.3; 1.5; 8–13.9
females (n = 16)	0.7; 0.2; 0.5–1.2	10.1; 1.2; 8–13.9
males and females (n = 53)	0.7; 0.2; 0.4–1.2	10.3; 1.4; 8–13.9
**Control**		
males (n = 25)	N/A	8.5; 1; 6.8–11.6
females (n = 35)	N/A	8.6; 1.4; 6.3–11.8
males and females (n = 60)	N/A	8.6; 1.2; 6.3–11.8

N/A: not applicable.

**Table 2:** Intergroup differences in shape space.

	Bratislava	Prague	Warsaw	Control
Bratislava	-	0.0667 *	0.0929 *	0.0993 *
Prague	0.002 **	-	0.0981 *	0.1278 *
Warsaw	<0.001 **	<0.001 **	-	0.0964 *
Control	<0.001 **	<0.001 **	<0.001 **	-

* Procrustes distance between respective groups; ** *p* value.

## Data Availability

Due to ethical limitations data will be available on a reasonable request from the corresponding author.

## Supplementary Materials

The following supporting information can be downloaded at: https://www.mdpi.com/article/10.3390/jcm12185985/s1, Supplementary Table S1: Non-trivial principal components in the whole sample.
