# Supplementary material for: The Slavcleft: A Three-Center Study of the Outcome of Treatment of Cleft Lip and Palate Considering Palatal Shape
Source: J Clin Med. 2023 Sep 15;12(18):5985. doi: 10.3390/jcm12185985 (PMC10531518; doi:10.3390/jcm12185985)
Supplement: Supplementary file 1 [file jcm-12-05985-s001.zip › jcm-2583561-supplementary.pdf]

Supplementary Table S1. Non-trivial principal components in the whole sample.

PC	Variance	% variance	broken-stick	Avg-Rnd	Rnd-Lambda
1	0.0034644624	23,9%	1.0%*	2.7399342E-004*	0.01000*
2	0.0026814402	18,5%	0.9%*	2.5654631E-004*	0.01000*
3	0.0015742568	10,9%	0.8%*	2.3856272E-004*	0.01000*
4	0.0010751396	7,4%	0.7%*	2.2769531E-004*	0.01000*
5	0.00076865929	5,3%	0.7%*	2.1958328E-004*	0.01000*
6	0.00067099196	4,6%	0.7%*	2.1382041E-004*	0.01000*
7	0.00052213226	3,6%	0.7%*	2.0856309E-004*	0.01000*
8	0.00044866398	3,1%	0.6%*	2.0404114E-004*	0.01000*
9	0.00033894868	2,3%	0.6%*	1.9905460E-004*	0.01000*
10	0.00030984105	2,1%	0.6%*	1.9546957E-004*	0.01000*
11	0.00026809605	1,8%	0.6%*	1.9171307E-004*	0.01000*
12	0.00022236808	1,5%	0.6%*	1.8776728E-004*	0.01000*
13	0.00019945744	1,4%	0.6%*	1.8465737E-004*	0.01000*
14	0.00017014075	1,2%	0.6%*	0.00018186965	1
15	0.0001394306	1,0%	0.5%*	0.00017869564	1
16	0.0001227475	0,8%	0.5%*	0.00017575226	1
17	0.00010510231	0,7%	0.5%*	0.00017266432	1
18	0.00010381862	0,7%	0.5%*	0.00017003086	1
19	0.000092748292	0,6%	0.5%*	0.0001679759	1
20	0.000073045027	0,5%	0.5%*	0.00016491115	1
	sum:	91.9%			

* denotes non-trivial PC.
